# Childhood Trauma and Subjective Symptoms of Inflammatory Bowel Disease Health-Related Quality of Life in the General Population: The Mediating Roles of Emotional Suppression and Cognitive Control

**DOI:** 10.3390/ijerph23081023

**Published:** 2026-08-05

**Authors:** Lisa Roque, Lorelei Van Hees, Bebiana Leonardo, Teresa Santos, Paulo Ferrajão

**Affiliations:** 1 Health Sciences Faculty, Universidade Europeia, 1500-210 Lisboa, Portugal; loreleivh@hotmail.com (L.V.H.); bebianauni.2024@gmail.com (B.L.); teresa.santos@universidadeeuropeia.pt (T.S.); paulo.ferrajao@universidadeeuropeia.pt (P.F.); 2 Research Centre in Sports Sciences, Health Sciences and Human Development (CIDESD), 5001-801 Vila Real, Portugal

**Keywords:** childhood trauma, emotional suppression, cognitive control, IBD health-related QoL, general population

## Abstract

**Highlights:**

**Public health relevance—how does this work relate to a public health issue?**

- Inflammatory bowel disease (IBD) affects millions worldwide, imposing substantial clinical consequences and mental health implications.
- Worldwide, IBD is entering a compounding prevalence period, which leads to accumulated prevalence over time and creates growing pressure on healthcare systems and economies, turning IBD into a global disease.

**Public health significance—why is this work of significance to public health?**

- Understanding these mechanisms can strengthen the development of early childhood support programs and resilience-focused interventions that may help reduce healthcare costs by preventing or reducing IBD symptom severity and long-term disease burden.
- This work highlights the importance of integrating psychological assessment into routine public health services.

**Public health implications—what are the key implications or messages for practitioners, policy makers and/or researchers in public health?**

- For practitioners, assessing these psychological processes offers a valuable window into patient risk profiles, enabling earlier identification of individuals more vulnerable to poor quality of life (QoL) due to maladaptive emotional and cognitive patterns.
- For researchers, general-population-based data collection on IBD, beyond clinical settings, is vital for informed resource allocation and the design of effective screening, prevention and public health interventions, which capture both diagnosed and undiagnosed cases.

**Abstract:**

Background: Inflammatory bowel disease (IBD) affects millions worldwide, imposing substantial clinical consequences and mental health implications. This burden leads to reduced quality of life (QoL) and growing pressure on healthcare systems and economies. This cross-sectional study examined the effects of childhood trauma on subjective symptoms of IBD health-related QoL, focusing on the mediating roles of emotional suppression and cognitive control, providing an integrative model for the general population. Methods: A sample of 402 Portuguese adults completed self-report measures, including the Childhood Trauma Questionnaire, the Emotion Regulation Questionnaire, the Cognitive Control and Flexibility Questionnaire, and the Short IBD Questionnaire. Multiple-step mediation analyses with bootstrapped confidence intervals tested indirect effects. Results: The two-step mediation effects indicated that childhood trauma was associated with subjective symptoms of IBD health-related QoL through both expressive suppression and cognitive control. Conclusions: These findings highlight the pivotal role of developmental, emotional and cognitive factors in understanding IBD health-related QoL in the general population, offering an opportunity to capture undiagnosed, mild, atypical, or intermittent cases, often discarded from most clinical studies. General-population-based data collection on IBD, beyond clinical settings, is vital for informed resource allocation and the design of effective screening, prevention and public health interventions that capture both diagnosed and undiagnosed cases.

## 1. Introduction

The global burden of inflammatory bowel diseases (IBDs) is profound and steadily increasing, advancing worldwide through defined epidemiological phases [1]. Many Western countries are entering a compounding prevalence period, in which the number of people living with this chronic condition is increasing rapidly, despite stable or declining new case rates. This pattern arises because incidence remains higher than mortality, and improved care prolongs patients’ survival rates, which leads to accumulated prevalence over time and growing pressure on healthcare systems and economies. Newly industrialized regions are expected to follow, intensifying global health, economic, and societal burdens by 2045, turning IBD into a global disease [2]. IBDs, such as Crohn’s disease or ulcerative colitis, are chronic, immune-mediated conditions characterized by an early onset (15–30 years), relapse and remission pattern, associated with persistent inflammation and structural damage of the gastrointestinal tract, including abdominal pain, changes in bowel habits (i.e., often alternating between diarrhea and constipation), blood in stool, rectal bleeding, and systemic complications (e.g., fatigue, weight loss, loss of appetite) [3]. Importantly, these disorders often severely impair patients’ quality of life (QoL), not only due to their physical manifestations but also due to their psychological and emotional toll [4]. Previous systematic reviews and meta-analyses have demonstrated that individuals with IBD experience poorer QoL compared to healthy individuals, both in children and adults [5]. QoL in individuals with IBD is influenced by a complex interplay of physical, psychological and social factors. Greater symptom burden and chronic pain are associated with increased fatigue, less physical activity and impaired daily functioning. Sexual health and fertility are significantly affected. Psychosocial well-being is often compromised due to IBD’s unpredictable nature, remitting and relapsing conditions, associated treatment-related burden and vigilant monitoring [6]. Psychological comorbidities, including anxiety and depression, contribute substantially to reduced QoL and predict a more complicated disease course [7].

IBD has been traditionally linked to multiple factors, such as genetic predisposition, nutritional deficiencies and lifestyle habits, such as smoking [8]. However, the understanding of health outcomes requires acknowledging intricate connections between environmental factors, psychological states and biological mechanisms within a biopsychosocial model [9]. A growing body of literature supports the existence of a bidirectional relationship between the central nervous system and the gastrointestinal tract, commonly referred to as the *gut–brain axis* [10]. This approach highlights how early stress, mental health (e.g., depression, anxiety), and physiological changes, including microbial dysbiosis and gut permeability, interact bidirectionally to influence IBD development [3]. Within this framework, childhood trauma has emerged as a critical factor in the etiology and exacerbation of gastrointestinal symptoms later in life [11,12,13]. Childhood trauma and adversity have been linked to chronic activation of the hypothalamic–pituitary–adrenal (HPA) axis, immune dysregulation, and heightened visceral sensitivity [3,10], all of which may contribute to the development or worsening of bowel inflammation and associated subjective symptoms. In parallel, emotional dysregulation, defined as difficulties in identifying, expressing, and managing emotional responses, has been consistently associated with both trauma exposure and poorer health outcomes across multiple domains, including chronic and systemic inflammation [14,15,16,17]. In this context, the roles of emotional and cognitive factors, such as emotional suppression and cognitive control, are gaining attention as potential mediators that link early trauma to systemic inflammation severity, gut microbiome composition and related impaired QoL [18,19,20].

### 1.1. The Role of Emotional Suppression

Emotional suppression refers to the deliberate inhibition of emotional expression, either positive or negative [21]. Though it may yield short-term social benefits and avoid immediate suffering, chronic use of suppression is considered dysfunctional, in that it is associated with adverse psychological and physiological outcomes, including increased stress and cortisol secretion, emotional discomfort, and an overall decrease in psychological well-being and the ability to feel pleasure [21,22,23]. In trauma-exposed populations, suppression is commonly used to avoid emotionally troubling memories or feelings [22]. In the context of IBD, research has shown that emotional suppression relates to both the severity of symptoms and impaired health-related QoL. This effect might stem from interfering with effective emotional regulation and greater internalization of stress, leading to disruptions in bidirectional gut–brain communication [14,18,19]. Compared to other emotion regulation strategies, such as cognitive reappraisal, expressive suppression may represent a more pertinent pathway between childhood trauma and subjective symptoms of IBD health-related QoL, as it is associated with a trauma-related pattern of inhibition. Unlike cognitive reappraisal, which involves adaptive modification of emotional experiences, expressive suppression primarily reduces the outward expression of emotions without necessarily attenuating internal emotional arousal [24]. Chronically, this can lead to poor psychological adjustment and altered stress responses, associated with chronic inflammation in clinical samples [22]. Thus, it is plausible to hypothesize that individuals with greater childhood trauma may engage in higher levels of emotional suppression, which in turn contributes to a lower quality of life due to exacerbated subjective symptoms of intestinal inflammation.

### 1.2. The Role of Cognitive Control

Cognitive control is a mental system that encompasses a set of high-level psychological processes that support goal-directed and adaptive behavior, including the selection of relevant information, inhibition of inappropriate responses, working memory, monitoring of ongoing behavior and flexible adjustment to changes in the environment [25]. These abilities are critical for coping with stress and managing intricate psychosocial environments. Early trauma exposure is known to moderate the association between inhibitory control and the anterior cingulate cortex stress reactivity [26] and impact cognitive control across the lifespan [27]. Reduced cognitive control has been associated with rumination and worrying [28], increased negative emotional reactivity [29], poorer emotional conflict adaptation [30], anxiety disorders, depression and severe mental illnesses [31], which may all amplify the distress associated with IBD symptoms and reduce perceived QoL. Thus, it is reasonable to suggest that reduced cognitive control may be associated with lower IBD health-related QoL, as it compromises executive functions related to emotion regulation and disease self-management [29,30,31]. Individuals with lower cognitive control may show more difficulties in regulating negative emotions, managing painful symptoms or implementing adaptive coping strategies, which may be associated with increased psychological and physiological distress, such as inflammation. These obstacles can negatively affect daily and social functioning, ultimately compromising health-related quality of life.

Most IBD studies rely on clinical populations, including hospital or specialty clinic patients, capturing primarily diagnosed, often severe, cases [32]. This approach misses undiagnosed, mild, atypical or intermittent disease, underestimating true prevalence, delaying early detection, and hindering accurate assessment of healthcare needs, economic impact, and societal burden [33]. We try to address this gap by analyzing the relationships between early childhood trauma exposure and the quality of life associated with symptoms of bowel inflammation, as well potential emotional and cognitive mediating factors (i.e., emotional suppression and cognitive control), in the general population. Collecting data on IBD in the general population, rather than only in clinical settings, is critical as it allows for better resource planning, targeted screening, prevention strategies, and public health interventions, ensuring that both diagnosed and undiagnosed individuals are considered in healthcare policies and research.

### 1.3. The Present Study

The present study seeks to advance our understanding of how childhood trauma may be associated with subjective symptoms of IBD health-related QoL in the general population. The overarching objective is to test a model in which childhood trauma has a direct negative effect on QoL associated with subjective symptoms of bowel inflammation and an indirect effect through heightened emotional suppression and reduced cognitive control.

In sum, the current study proposes the following hypotheses:

1. There is a direct effect between childhood trauma and subjective symptoms of IBD health-related QoL in the general population; specifically, greater childhood trauma will be associated with lower QoL.
2. There is an indirect effect of emotional suppression on the relationship between childhood trauma and subjective symptoms of IBD health-related QoL in the general population, such that greater childhood trauma is associated with greater emotional suppression, which in turn is associated with lower QoL.
3. There is an indirect effect of cognitive control on the relationship between childhood trauma and subjective symptoms of IBD health-related QoL in the general population, such that greater childhood trauma is associated with lower cognitive control, which in turn is associated with lower QoL.

## 2. Materials and Methods

### 2.1. Participants

The sample consisted of 402 Portuguese adults, all over 18 years of age (M = 35.44 years, SD = 13.18; range = 18–78). Women represented a substantial majority of the sample, indicating a marked overrepresentation of female participants. Regarding marital status, most individuals were single, whereas 27.1% were married or cohabiting, and only a small proportion were divorced or widowed. In terms of educational attainment, the sample was generally highly educated, while 31.1% had completed 12 years of schooling, and only a minority reported lower levels of education. Socioeconomic status followed a similar pattern of concentration in the middle categories, with most participants identifying as middle class, 27.1% as middle–low, and only a small proportion identifying as belonging to the middle–high or high socioeconomic classes (Table 1).

The estimation of sample size for multiple-step mediation methodology was conducted. This procedure was performed with an expected RMSEA of 0.05, 8 observed variables, a significance level of 0.05, and a sample size power of 95%, which resulted in an estimated sample size of 328 participants [34].

### 2.2. Procedure

A cross-sectional study with convenience sampling was conducted. Data collection occurred between January and May 2025. The participants were given access to the questionnaire through a link and a QR code shared on social media. All participants gave their informed consent before responding, and all questionnaires were kept anonymous. The study was approved by the Ethical Review Board of the University.

Presently, no Portuguese versions exist for three of the measures used in this study: the *Emotion Regulation Questionnaire*, the *Cognitive Control and Flexibility Questionnaire*, and the *Short Inflammatory Bowel Disease Questionnaire*. The instruments were adapted by at least 3 members of the research team using a forward–backward translation procedure to ensure semantic, conceptual, and cultural equivalence. The process included independent forward translation, reconciliation, blinded back-translation and expert review, according to the best scientific practices [35]. Prior to data analysis, the adequacy of the factorial solution for the three measures was examined using confirmatory factor analysis (CFA). Model fit was evaluated using the following criteria: (a) a non-significant χ^2^ test; (b) comparative fit index (CFI), normed fit index (NFI), and Tucker–Lewis index (TLI) values greater than 0.95; and (c) root mean square error of approximation (RMSEA) and standardized root mean square residual (SRMR) values between 0.00 and 0.08. Given the sensitivity of the χ^2^ test to sample size, the ratio of χ^2^ to degrees of freedom (χ^2^/df) was also examined, with values between 1 and 5 indicating an acceptable fit between the hypothesized model and the observed data [36]. Factor loadings were inspected to ensure that each item contributed meaningfully to its respective latent construct. In accordance with established guidelines, factor loadings greater than 0.30 were considered acceptable, reflecting a minimum level of practical significance [37]. Internal consistency for all scales was assessed using Cronbach’s alpha coefficients.

### 2.3. Measures

*The Childhood Trauma Questionnaire—Short Form* (CTQ-SF) [38], Portuguese version by [39], is a measure that retrospectively assesses an individual’s experiences of childhood maltreatment. The CTQ-SF is a 28-item self-report measure to which respondents answer to each experience on a 5-point Likert scale, ranging from (1) never true to (5) very often true. This measure provides a global index of childhood maltreatment (global score), including five subscales: emotional abuse, physical abuse, sexual abuse, emotional neglect, and physical neglect. In this study, the global score of childhood trauma, which was calculated by adding up all the items that compose the scale, was analyzed. The total score ranges from 25 to 125. Internal consistency of the global score was good, with Cronbach’s α = 0.82.

*The Emotion Regulation Questionnaire* (ERQ) [24] is a measure that assesses an individuals’ tendency to regulate their emotions. The ERQ is a 10-item self-report measure to which respondents answer to each item on a 7-point Likert scale, ranging from (1) strongly disagree to (7) strongly agree. The measure provides two subscales reflecting two distinct emotion regulation strategies: cognitive reappraisal and expressive suppression. For the purposes of this study, analyses were restricted to the expressive suppression subscale. The score for the expressive suppression subscale was obtained by summing the responses to all items corresponding to the scale; for the expressive suppression subscale, total scores range from 4 to 28, with higher values indicating greater reliance on expressive suppression. The factorial structure of the instrument was examined through CFA. The results indicated that the scale replicated the factor structure of the original version, demonstrating an adequate model fit within the present sample (χ2 (27) = 22.03, *p* = 0.74; ratio of chi square to degrees of freedom = 0.82; NFI = 0.99; CFI = 0.99; TLI = 0.99; RMSEA = 0.01; SMSR = 0.02). All items exhibited factor loadings greater than 0.30 on their respective factors. Internal consistency of both subscales was satisfactory-to-good, with a Cronbach’s α of 0.78 for emotional suppression and 0.86 for cognitive reappraisal.

*The Cognitive Control and Flexibility Questionnaire* (CCFQ) [40] is a measure that assesses an individual’s perceived capacity to regulate cognitive processes and to adapt flexibly to changing situational demands. The CCFQ is an 18-item self-report measure to which respondents answer to each item on a 7-point Likert scale, ranging from (1) strongly disagree to (7) strongly agree, indicating the degree to which the statements reflect their typical cognitive functioning. It evaluates two primary dimensions: cognitive control and cognitive flexibility. The scores for the two subscales were derived by calculating the mean of the summed items corresponding to each subscale. Total scores for both subscales can range from 1 to 7, with higher scores indicating greater perceived cognitive control and flexibility. In the absence of an existing Portuguese version of this instrument, the scale version produced through the translation procedure was presented to the participants. The factorial structure of the instrument was examined through CFA. The results indicated that the scale replicated the factor structure of the original version, demonstrating an adequate model fit within the present sample (χ2 (89) = 109.85, *p* = 0.07; ratio of chi square to degrees of freedom =1.23; NFI = 0.98; CFI = 0.99; TLI = 0.99; RMSEA = 0.02; SMSR = 0.03). All items exhibited factor loadings greater than 0.30 on their respective factors. Internal consistency of both subscales was good, with a Cronbach’s α of 0.89 for cognitive control and 0.92 for cognitive flexibility. In this study, only the cognitive control subscale was examined and included in the analyses.

*The Short Inflammatory Bowel Disease Questionnaire* (SIBDQ) [4,41] is an instrument designed to assess health-related QoL in individuals with IBD. In this study, the measure was slightly adapted to ensure that the content of the items was appropriate for use in the general population rather than exclusively for individuals clinically diagnosed with IBD, for whom the instrument was originally developed. The adaptation consisted of replacing “bowel-problem”, used in the original version, with “symptoms of bowel pain/discomfort”. The SIBDQ is a 10-item self-report measure to which respondents answer to each item on a 7-point Likert scale, and it includes two response formats: one ranging from (1) worst to (7) best and another ranging from (1) never to (7) always. This measure provides a global score of QoL and scores on four subscales: Bowel Symptoms, Emotional Health, Social Function, and Systemic Symptoms. In this study, the global score of the measure was analyzed, which was calculated by adding up all the items that comprise the SIBDQ. The global score ranges from 7 to 70, with higher scores indicating better QoL. An SIBDQ score of less than 50 was considered to indicate a poor QoL. The one-factor structure of the scale was examined using CFA. The results supported the adequacy of the scale’s unidimensional structure, with Item 3 coded in the reverse direction (χ2 (26) = 57.26, *p* < 0.01; ratio of chi square to degrees of freedom = 2.20; NFI = 0.97; CFI = 0.99; TLI = 0.97; RMSEA = 0.05; SMSR = 0.04). All items exhibited factor loadings greater than 0.30 on their respective factors. Internal consistency of the global score was good, with a Cronbach’s α of 0.88.

The sociodemographic questionnaire included items aimed at collecting information on participants’ sex, age, marital status, level of education, and socioeconomic status.

### 2.4. Data Analysis

Descriptive statistics, including measures of central tendency and dispersion (means and standard deviations), as well as range indicators (minimum and maximum values), were calculated for all study variables. Bivariate associations among variables were assessed using Pearson’s correlation analysis, with correlation coefficients classified as strong (±0.70–1.00), moderate (±0.30–0.69), or weak (≤±0.29). All statistical analyses were performed using IBM SPSS Statistics for Windows (Version 29).

We examined the proposed indirect effects of childhood maltreatment on IBD health-related QoL through expressive suppression and cognitive control using a multistep mediation analysis [42]. The models were evaluated using structural equation modeling (SEM). The dataset contained no missing values.

Prior to conducting the structural equation modeling analyses, the underlying statistical assumptions, including multivariate normality, linearity, absence of multicollinearity, and adequacy of model identification, were systematically evaluated to ensure the appropriateness and robustness of the estimated models [36,37]. The models were assessed using SEM implemented in AMOS version 29, with parameters estimated via the maximum likelihood method in accordance with the approach described by Hoyle and Smith [43].

This study investigated both the direct effects of childhood maltreatment global score on IBD health-related QoL and their indirect effects mediated by expressive suppression and cognitive flexibility. The examination of indirect effects was conducted in two sequential phases. A multiple-step mediation approach was employed to test the hypothesized serial mediation model, with indirect effects estimated using bootstrapped confidence intervals [44]. All variables were modeled as observed indicators.

First, to test the multiple mediation hypotheses, we examined (a) the direct effects of the global childhood maltreatment score on IBD health-related QoL and (b) the indirect effects of childhood maltreatment on IBD health-related QoL through expressive suppression and cognitive control. Second, to evaluate the integrated serial mediation model, we examined (a) the direct effects of childhood maltreatment on IBD health-related QoL; (b) the indirect effects through expressive suppression; (c) the indirect effects through cognitive control; and (d) the indirect effects via a two-stage serial mediation pathway involving expressive suppression followed by cognitive control.

Model adequacy was evaluated using established structural equation modeling criteria, including: (a) a non-significant chi-square (χ^2^) statistic; (b) comparative fit index (CFI), normed fit index (NFI), and Tucker–Lewis Index (TLI) values greater than 0.95; and (c) root mean square error of approximation (RMSEA) and standardized root mean square residual (SRMR) values below 0.08. Because both models are non-nested, comparative fit was assessed using the Akaike Information Criterion (AIC) and Bayesian Information Criterion (BIC), with lower values indicating better fit [45]. Indirect effects were examined using a bootstrapping approach with 5000 resamples, as recommended by Preacher and Hayes [44]. Bias-corrected and accelerated 95% confidence intervals (CIs) were calculated for both single- and two-step mediation effects. Effects were considered statistically significant if the corresponding confidence interval did not encompass zero. There were no missing data.

## 3. Results

### 3.1. Intercorrelations Between Study Variables

As shown in Table 2, the childhood trauma score was weakly positively associated with expressive suppression and moderately negatively associated with both cognitive control and IBD health-related QoL. Expressive suppression was weakly negatively associated with both cognitive control and IBD health-related QoL. Finally, cognitive control was moderately positively associated with IBD health-related QoL.

### 3.2. Multiple Mediation Analyses

The assumptions required for the application of structural equation modeling were systematically evaluated prior to model estimation. Specifically, the data were examined for normality, linearity, absence of multicollinearity, and adequacy of sample size, as well as for the presence of outliers and missing values. The results of these diagnostic analyses indicated that all relevant assumptions were satisfactorily met, supporting the appropriateness of using structural equation modeling for the subsequent analyses.

**Figure 1:**
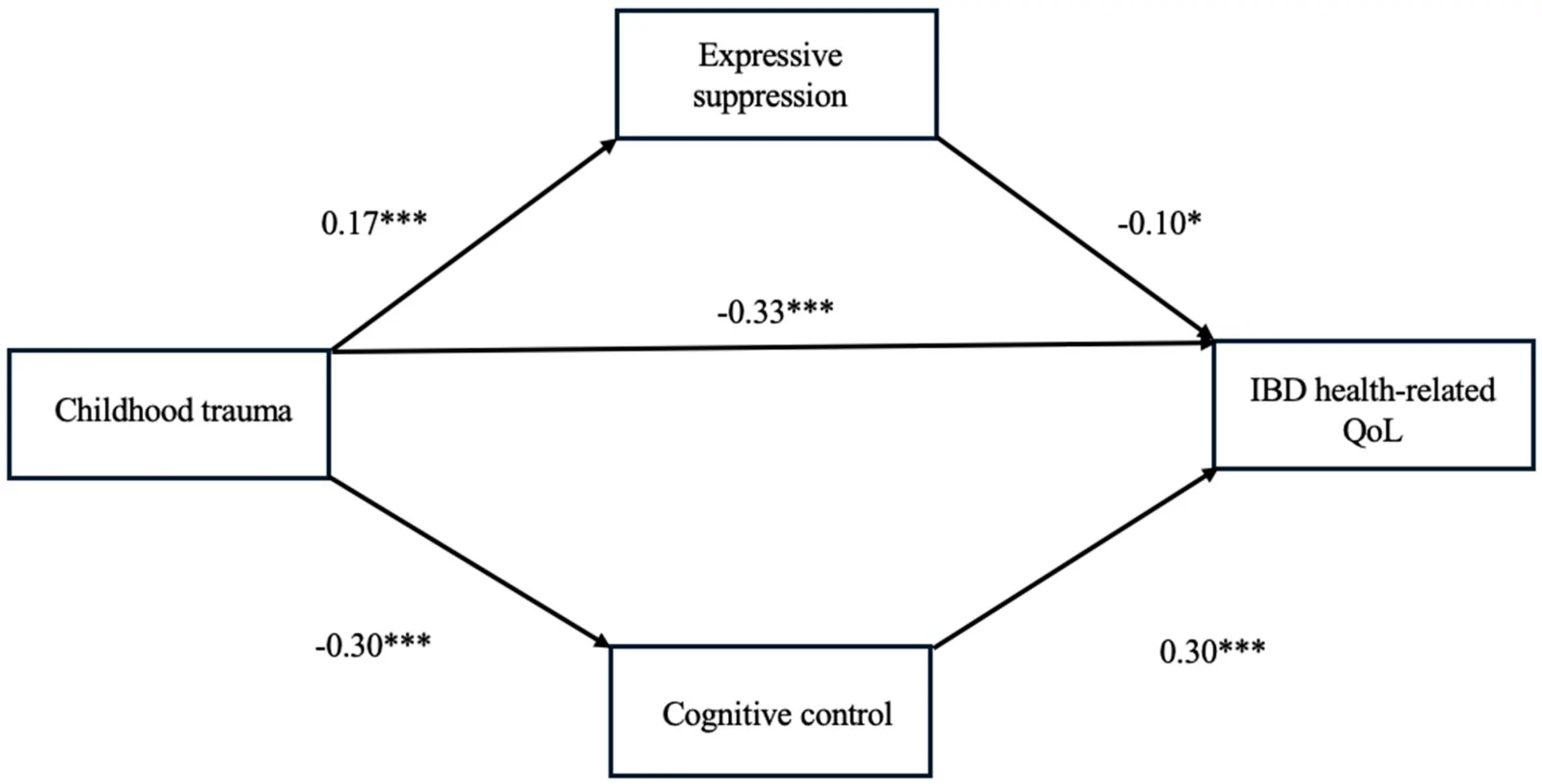
Multiple mediation model for IBD health-related QoL by childhood trauma through expressive suppression and cognitive control. Rectangles indicate measured variables. Standardized maximum likelihood parameters are used. Unidirectional arrows depict hypothesized directional links. Standardized regression coefficients are presented. * *p* < 0.05, *** *p* < 0.001.

The multiple mediation model that tested the direct paths from childhood maltreatment score to IBD health-related QoL and indirect paths through expressive suppression and cognitive control showed a good fit to the observed data (χ2 (1) = 0.14, *p* = 0.71; NFI = 0.99; CFI = 0.99; TLI = 0.99; RMSEA = 0.01; SMSR = 0.01; AIC = 33.79; BIC = 162.38). Unstandardized and standardized coefficients, together with bootstrap estimates, are reported in Table 3, whereas the corresponding unstandardized coefficients are illustrated in Figure 1. The direct path from childhood trauma to IBD health-related QoL was statistically significant and of moderate magnitude (*β* = −0.33), indicating that greater exposure to childhood trauma was associated with poorer IBD health-related QoL.

The indirect effects of childhood trauma on inflammatory bowel disease-related quality of life through expressive suppression and cognitive control were statistically significant. The standardized indirect effect through expressive suppression was small (*β* = −0.17), whereas the indirect effect through cognitive control was of moderate magnitude (*β* = −0.30). Specifically, higher levels of childhood trauma were associated with greater use of expressive suppression and lower levels of cognitive control, which, in turn, were associated with poorer IBD health-related QoL.

### 3.3. Analysis of Serial Mediation

The integrated serial mediation model that tested the direct effects of different forms of childhood trauma on IBD health-related QoL, as well as the indirect effects of childhood trauma on IBD health-related QoL, via a two-stage mediation pathway involving expressive suppression followed by cognitive control showed a good fit to the observed data (χ2 (1) = 0.33, *p* = 0.57; NFI = 0.99; CFI = 0.99; TLI = 0.99; RMSEA = 0.01; SMSR = 0.01; AIC = 28.35; BIC = 154.68). The results indicated that the integrated serial mediation model demonstrated superior model fit compared to the multiple mediation model, as evidenced by lower AIC and BIC values.

**Figure 2:**
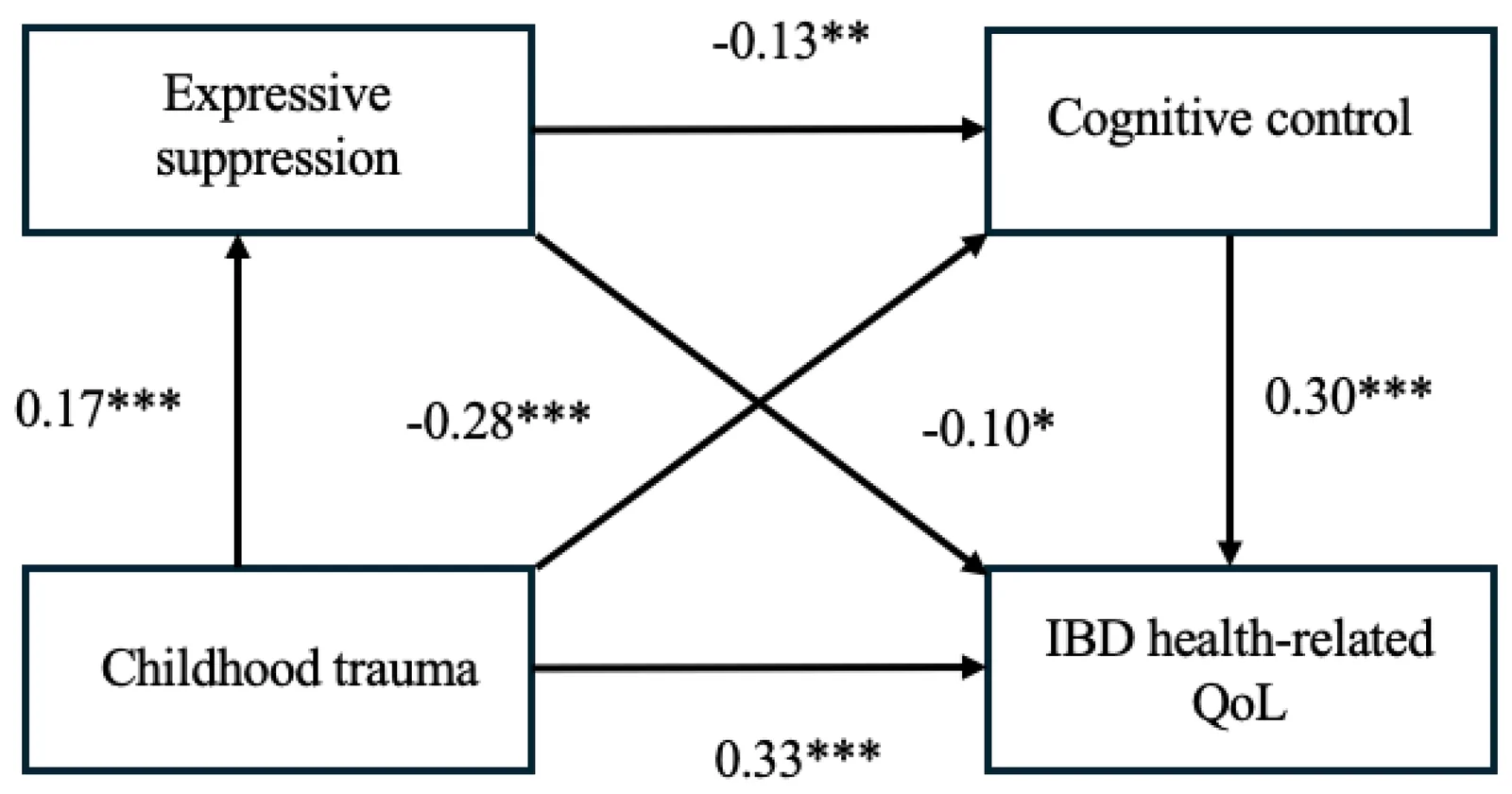
Integrated serial mediation model for IBD health-related QoL by childhood trauma through expressive suppression followed by cognitive control. Rectangles indicate measured variables. Standardized maximum likelihood parameters are used. Unidirectional arrows depict hypothesized directional links. Standardized regression coefficients are presented. * *p* < 0.05, ** *p* < 0.01, *** *p* < 0.001.

Unstandardized and standardized coefficients, together with bootstrap estimates, are reported in Table 4, whereas the corresponding unstandardized coefficients are illustrated in Figure 2. The direct path from childhood trauma to IBD health-related QoL remained statistically significant and of moderate magnitude (*β* = −0.33), indicating that greater exposure to childhood trauma was associated with poorer IBD health-related QoL.

Although the indirect effect through expressive suppression remained statistically significant, its standardized magnitude was relatively small (*β* = −0.17), suggesting that expressive suppression accounted for a limited proportion of the association between childhood trauma and IBD health-related QoL. The results indicated that higher levels of childhood trauma were significantly associated with higher levels of expressive suppression, which were associated with lower levels of IBD health-related QoL. Likewise, the indirect effect through cognitive control remained statistically significant and was of moderate magnitude (*β* = −0.28), suggesting that cognitive control accounted for a meaningful proportion of the association between childhood trauma and IBD health-related QoL. Higher levels of childhood trauma were associated with lower levels of cognitive control, which were associated with lower levels of IBD health-related QoL.

Finally, although the two-step indirect effect through expressive suppression and cognitive control remained statistically significant, its standardized magnitude was small (*β* = −0.13), suggesting that this sequential mediation pathway explained only a modest proportion of the association between childhood trauma and IBD health-related QoL. Specifically, higher levels of childhood trauma were associated with higher levels of expressive suppression, which were associated with lower levels of cognitive control, which in turn were associated with lower levels of IBD health-related QoL.

## 4. Discussion

IBD affects millions of people daily, placing significant physical, emotional, and economic burdens on individuals and healthcare systems worldwide. Symptoms commonly associated with it, including diarrhea, abdominal pain, and fatigue, are also frequently experienced in the general population, even without a formal diagnosis. Mild, intermittent and nonspecific symptoms may initially be overlooked or attributed to other functional gastrointestinal disorders before underlying intestinal inflammation is recognized. In that sense, collecting data on IBD in the general population, rather than only in clinical settings, is key. In the context of this study, studying the relationship between early trauma and IBD symptoms is crucial for understanding how psychological experiences influence physical health. Early-life stress can shape the immune system and gut–brain communication, potentially exacerbating IBD symptoms and diminishing health-related QoL, both in diagnosed and non-diagnosed populations. Exploring cognitive and emotional factors, such as emotional suppression and cognitive control, may clarify why some individuals are more vulnerable to developing IBD symptomatology, even before being properly diagnosed by a professional doctor. The present results revealed a direct relationship between increased childhood trauma and lower health-related QoL associated with inflammatory bowel disease symptoms in the general population, supporting existing evidence that individuals with traumatic backgrounds are particularly at risk for adverse IBD-related outcomes [3,12,13]. The findings also showed a significant indirect effect of emotional suppression on the relationship between childhood trauma and QoL associated with subjective symptoms of IBD symptoms in the general population. Greater exposure to childhood trauma was associated with higher emotional suppression, which predicted lower QoL. These results align with the prior literature indicating that childhood trauma interferes with the brain’s automatic regulation of emotional responses, both in neural activity and behavior [46]. This disruption of emotional processing then explains how exposure to early-life adversity is associated with poor physical health in adulthood, including prospective changes in systemic inflammation and inflammatory biomarkers, as well as the regulation and composition of the gut microbiome [14,18,19]. These results are aligned with previous work with clinical samples, proposing that effects of psychological stress on IBD symptomatology may be explained by its interference with adaptive emotional regulation, increased internalization of stress, and the disruption of gut–brain axis homeostasis [47].

The current findings also revealed an indirect effect of cognitive control on the relationship between childhood trauma and QoL associated with subjective symptoms of IBD in the general population. Greater exposure to childhood trauma was associated with lower cognitive control, which predicted lower QoL. This finding is consistent with prior evidence collected in clinical samples, indicating that acute psychological stress modulates neural and behavioral substrates of cognitive control [48]. Impairments in cognitive control are linked to diminished capacity for flexible, goal-oriented behavior [25], greater rumination, worrying and negative emotionality [28,29], and increased pro-inflammatory cytokine reactivity to stress [20], which can intensify the discomfort associated with IBD symptoms and diminish overall perceived QoL. Furthermore, these results are aligned with recent findings highlighting the bidirectional communication between gastrointestinal vagus nerve signaling and neurocognitive processing in shaping adaptive behavioral responses following severe and psychological stress [47,49].

Although the observed two-step indirect effect through expressive suppression and cognitive control remained statistically significant, its standardized magnitude was small. These pathways constitute modest contributions rather than primary explanatory factors. Small effect sizes are common in biopsychosocial models of chronic diseases such as IBD, where outcomes are associated with multiple interacting psychological, environmental and biological pathways. From a clinical perspective, the small magnitude of these effects does not imply limited pertinence and implications at the population level, as they may influence disease management, treatment involvement, and even physiological stress responses and immune regulation, which should be addressed in future studies. Therefore, the practical significance of these results resides in identifying emotional and cognitive psychological processes that may contribute to a wider and complex interplay of risk and protective factors. For clinicians, assessing these psychological processes offers a valuable window into patient risk profiles, enabling earlier identification of individuals more vulnerable to poor QoL due to maladaptive emotional and cognitive patterns. Targeted interventions, such as cognitive–behavioral therapy aimed at improving emotional regulation and enhancing cognitive control, could significantly alleviate symptom severity and improve functional outcomes. Moreover, understanding these mediating factors allows for personalized treatment plans, optimizing both psychological and medical approaches to care.

Finally, the predominantly female and highly educated composition of our sample may limit the generalizability of these findings. Women with IBD show higher psychological burden and affective disorders, including levels of anxiety and depressive symptoms, compared to men, which may also be associated with differences in emotion regulation strategies [50,51]. On the other hand, higher education may increase disease self-management behaviors, but it can also enhance symptom recognition and reporting. Future studies should examine these relationships in more diverse and representative cohorts and include gender and other contextual factors as potential moderators in integrative models exploring the relationships between childhood trauma and IBD symptoms or health-related QoL.

## 5. Limitations and Future Research

Although the current study offers important insights into the pathways linking early trauma and QoL associated with IBD symptoms in the general population, there are several factors that should be considered. The cross-sectional nature of the study and its reliance on self-report data and recall-based trauma reports restricts the capacity to determine causal relationships or control social desirability and respondent fatigue. Incorporating longitudinal and multidisciplinary methods and approaches could strengthen future research, including inflammatory biomarkers, gut microbiome composition and cortisol reactivity. Finally, the absence of adjustment for potential demographic and clinical confounding variables (e.g., age, gender, socioeconomic status, mental health symptoms, disease-related characteristics, and comorbid chronic conditions) limits the estimation of the independent associations of interest, and residual confounding cannot be excluded. Future studies should incorporate relevant covariates into multivariable SEM analyses to improve the robustness and generalizability of the findings. Additionally, future research analyzing and comparing data from both general and clinical populations would be particularly relevant, ensuring that both diagnosed and undiagnosed individuals (e.g., mild, atypical, intermittent) are considered in healthcare policies and research.

## 6. Conclusions

This study illustrates how childhood trauma influences IBD health-related QoL in the general population, highlighting the pivotal role of emotional suppression and cognitive control. Higher levels of childhood trauma were associated with higher levels of expressive suppression and lower levels of cognitive control, which were associated with lower levels of IBD health-related QoL. Understanding these pathways is critical not only for elucidating the long-term consequences of childhood adversity but also for informing therapeutic approaches aimed at enhancing emotion regulation and executive functioning in trauma-exposed populations with IBD complaints, even without proper formal diagnosis. Moreover, by focusing on symptom perception and QoL, as opposed to purely physiological markers, this study aligns with patient-centered models of care and public health approaches that prioritize mental and emotional well-being. Furthermore, general-population-based data collection on IBD health-related QoL is crucial in ensuring that both diagnosed and undiagnosed individuals are considered in effective prevention and future healthcare policies and research. Such data also allows for a better distinction between true risk factors and correlates of healthcare-seeking behaviors, strengthening and widening the public health pertinence and real prevalence of bowel inflammation multidisciplinary research.

## Figures and Tables

**Table 1:** Sample demographic characteristics.

	*n*	%
**Gender**		
Female	313	77.9
Male	89	22.1
**Marital status**		
Single	266	66.2
Married or cohabitation	109	27.1
Divorced	23	5.7
Widowed	4	1.0
**Education**		
Less than nine years of education	3	0.7
Nine years of education	15	3.8
12 years of education	125	31.1
Higher education	259	64.4
**Socioeconomic status**		
Middle–low	109	27.1
Middle	266	66.2
Middle–high	23	5.7
High	4	1.0

**Table 2:** Correlation matrix and descriptive statistics of study variables.

Variables	1.	2.	3.	4.
1. Childhood trauma global score	-	0.17 ***	−0.30 ***	−0.44 ***
2. Expressive suppression		-	−0.18 ***	−0.21 ***
3. Cognitive control			-	0.42
4. IBD health-related QoL				-
**Descriptive statistics**				
Mean	38.63	15.41	3.83	46.42
Standard deviation	13.53	5.27	1.22	9.13
Minimum	25	4	1	19
Maximum	97	28	7	64

Note. *** *p* < 0.001.

**Table 3:** Bootstrapped point estimates and 95% confidence intervals for direct and indirect effects in a multiple mediation model predicting IBD health-related QoL from childhood trauma global score via expressive suppression and cognitive control.

	B	B 95% CI (Lower, Upper)	β	B 95% CI (Lower, Upper)	*p*
Direct effect	−0.26	(−0.33, −0.19)	−0.33	(−0.39, −0.27)	0.001
Indirect effect through expressive suppression	−0.07	(−0.10, −0.03)	−0.17	(−0.19, −0.15)	0.001
Indirect effect through cognitive control	−0.03	(−0.04, −0.02)	−0.30	(−0.33, −0.27)	0.001

Note. B = unstandardized estimates; β = standardized estimates; CI = confidence intervals; confidence intervals that do not include 0 (null association) are significant.

**Table 4:** Bootstrapped point estimates and 95% confidence intervals for direct and indirect effects in an integrated serial mediation model predicting IBD health-related QoL from childhood trauma global score via expressive suppression and cognitive control.

	B	B 95% CI (Lower, Upper)	β	B 95% CI (Lower, Upper)	*p*
Direct effect	−0.26	(−0.33, −0.19)	−0.33	(−0.39, −0.27)	0.001
Indirect effect through expressive suppression	−0.07	(−0.10, −0.03)	−0.17	(−0.19, −0.15)	0.001
Indirect effect through cognitive control	−0.03	(−0.04, −0.02)	−0.28	(−0.32, −0.24)	0.001
Indirect effect through expressive suppression and cognitive control	−0.03	(−0.05, −0.01)	−0.13	(−0.17, −0.09)	0.006

Note. B = unstandardized estimates; β = standardized estimates; CI = confidence intervals; confidence intervals that do not include 0 (null association) are significant.

## Data Availability

The data that support the findings of this study are available on request from the corresponding author. The data are not publicly available due to privacy or ethical restrictions.

## References

[1] Kaplan G.G., Windsor J.W. (2021). The four epidemiological stages in the global evolution of inflammatory bowel disease. Nat. Rev. Gastroenterol. Hepatol..

[2] Kaplan G.G. (2025). The global burden of inflammatory bowel disease: From 2025 to 2045. Nat. Rev. Gastroenterol. Hepatol..

[3] Bonaz B., Sinniger V., Pellissier S. (2024). Role of stress and early-life stress in the pathogeny of inflammatory bowel disease. Front. Neurosci..

[4] Roseira J., Sousa H.T., Marreiros A., Contente L.F., Magro F. (2021). Short Inflammatory Bowel Disease Questionnaire: Translation and validation to the Portuguese language. Health Qual. Life Outcomes.

[5] Knowles S.R., Graff L.A., Wilding H., Hewitt C., Keefer L., Mikocka-Walus A. (2018). Quality of life in inflammatory bowel disease: A systematic review and meta-analyses—Part I. Inflamm. Bowel Dis..

[6] Khan S., Sebastian S.A., Parmar M.P., Ghadge N., Padda I., Keshta A.S., Minhaz N., Patel A. (2024). Factors influencing the quality of life in inflammatory bowel disease: A comprehensive review. Dis.-A-Mon..

[7] Swaminathan A., Fan D., Borichevsky G.M., Mules T.C., Hirschfeld E., Frampton C.M., Day A.S., Siegel C.A., Gearry R.B. (2022). The disease severity index for inflammatory bowel disease is associated with psychological symptoms and quality of life, and predicts a more complicated disease course. Aliment. Pharmacol. Ther..

[8] Fakhoury M., Negrulj R., Mooranian A., Al-Salami H. (2014). Inflammatory bowel disease: Clinical aspects and treatments. J. Inflamm. Res..

[9] O’Connor D.B., Thayer J.F., Vedhara K. (2021). Stress and health: A review of psychobiological processes. Annu. Rev. Psychol..

[10] Bernstein C.N. (2017). The brain-gut axis and stress in inflammatory bowel disease. Gastroenterol. Clin..

[11] Fuller-Thomson E., West K.J., Sulman J., Baird S.L. (2015). Childhood maltreatment is associated with ulcerative colitis but not Crohn’s disease: Findings from a population-based study. Inflamm. Bowel Dis..

[12] Gnat L., Mihajlovic V., Jones K., Tripp D.A. (2023). Differentiating childhood traumas in inflammatory Bowel disease. J. Can. Assoc. Gastroenterol..

[13] Minjoz S., Sinniger V., Hot P., Bonaz B., Pellissier S. (2023). The burden of early life stress in chronic inflammatory bowel diseases. J. Health Psychol..

[14] Jones E.J., Marsland A.L., Gianaros P.J. (2023). Do trait-level emotion regulation strategies moderate associations between retrospective reports of childhood trauma and prospective changes in systemic inflammation?. Stress Health.

[15] Mathur A., Li J.C., Lipitz S.R., Graham-Engeland J.E. (2022). Emotion regulation as a pathway connecting early life adversity and inflammation in adulthood: A conceptual framework. Advers. Resil. Sci..

[16] Moriarity D.P., Grehl M.M., Walsh R.F., Roos L.G., Slavich G.M., Alloy L.B. (2023). A systematic review of associations between emotion regulation characteristics and inflammation. Neurosci. Biobehav. Rev..

[17] Yuan J.P., Ho T.C., Coury S.M., Chahal R., Colich N.L., Gotlib I.H. (2022). Early life stress, systemic inflammation, and neural correlates of implicit emotion regulation in adolescents. Brain Behav. Immun..

[18] Ke S., Guimond A.J., Tworoger S.S., Huang T., Chan A.T., Liu Y.Y., Kubzansky L.D. (2023). Gut feelings: Associations of emotions and emotion regulation with the gut microbiome in women. Psychol. Med..

[19] Ospina L.H., Beck-Felts K., Ifrah C., Lister A., Messer S., Russo S.J., Gross J.J., Kimhy D. (2022). Inflammation and emotion regulation: Findings from the MIDUS II study. Brain Behav. Immun.-Health.

[20] Shields G.S., Kuchenbecker S.Y., Pressman S.D., Sumida K.D., Slavich G.M. (2016). Better cognitive control of emotional information is associated with reduced pro-inflammatory cytokine reactivity to emotional stress. Stress.

[21] Gross J.J., Ford B.Q. (2024). Handbook of Emotion Regulation.

[22] Hoffman S.N., Stein M.B., Taylor C.T. (2023). Childhood trauma predicts positive expressive suppression during social affiliation in adults with anxiety and/or depression: Implications for social functioning. Behav. Ther..

[23] Otto L.R., Sin N.L., Almeida D.M., Sloan R.P. (2018). Trait emotion regulation strategies and diurnal cortisol profiles in healthy adults. Health Psychol..

[24] Gross J.J., John O.P. (2003). Individual differences in two emotion regulation processes: Implications for affect, relationships, and well-being. J. Personal. Soc. Psychol..

[25] Badre D. (2025). Cognitive control. Annu. Rev. Psychol..

[26] Zhai Z.W., Yip S.W., Lacadie C.M., Sinha R., Mayes L.C., Potenza M.N. (2019). Childhood trauma moderates inhibitory control and anterior cingulate cortex activation during stress. NeuroImage.

[27] Rahapsari S., Levita L. (2025). The impact of adverse childhood experiences on cognitive control across the lifespan: A systematic review and meta-analysis of prospective studies. Trauma Violence Abus..

[28] Beckwé M., Deroost N., Koster E.H., De Lissnyder E., De Raedt R. (2014). Worrying and rumination are both associated with reduced cognitive control. Psychol. Res..

[29] Rónai L., Hann F., Kéri S., Ettinger U., Polner B. (2024). Emotions under control? Better cognitive control is associated with reduced negative emotionality but increased negative emotional reactivity within individuals. Behav. Res. Ther..

[30] Hantke N.C., Gyurak A., Van Moorleghem K., Waring J.D., Adamson M.M., O’Hara R., Beaudreau S.A. (2017). Disentangling cognition and emotion in older adults: The role of cognitive control and mental health in emotional conflict adaptation. Int. J. Geriatr. Psychiatry.

[31] Alker L. (2025). Cognitive control: Modeling the impact on mental health. Front. Psychol..

[32] Buie M.J., Quan J., Windsor J.W., Coward S., Hansen T.M., King J.A., Kotze P.G., Gearry R.B., Ng S.C., Mak J.W.Y. (2023). Global hospitalization trends for Crohn’s disease and ulcerative colitis in the 21st century: A systematic review with temporal analyses. Clin. Gastroenterol. Hepatol..

[33] Jayasooriya N., Baillie S., Blackwell J., Bottle A., Petersen I., Creese H., Saxena S., Pollok R.C., POP-IBD Study Group (2023). Systematic review with meta-analysis: Time to diagnosis and the impact of delayed diagnosis on clinical outcomes in inflammatory bowel disease. Aliment. Pharmacol. Ther..

[34] Kim K.H. (2005). The relation among fit Indexes, power, and sample size in Structural Equation Modeling. Struct. Equ. Model..

[35] Hambleton R.K., Patsula L. (1998). Adapting tests for use in multiple languages and cultures. Soc. Indic. Res..

[36] Kline R.B. (2016). Principles and Practice of Structural Equation Modeling.

[37] Hair J.F., Black W.C., Babin B.J., Anderson R.E. (2010). Multivariate Data Analysis.

[38] Bernstein D.P., Stein J.A., Newcomb M.D., Walker E., Pogge D., Ahluvalia T., Stokes J., Handelsman L., Medrano M., Desmond D. (2003). Development and validation of a brief screening version of the Childhood Trauma Questionnaire. Child Abus. Negl..

[39] Dias A., Sales L., Carvalho A., Castro-Vale I., Kleber R., Mota-Cardoso R. (2014). Estudo das propriedades psicométricas do Questionário de Trauma de Infância—Versão Breve (CTQ-SF) numa amostra portuguesa não clínica [Study of the psychometric properties of the Childhood Trauma Questionnaire—Short Form (CTQ-SF) in a non-clinical Portuguese sample]. Laboratório Psicol..

[40] Gabrys R.L., Tabri N., Anisman H., Matheson K. (2018). Cognitive control and flexibility in the context of stress and depressive symptoms: The Cognitive Control and Flexibility Questionnaire. Front. Psychol..

[41] Irvine E.J., Zhou Q., Thompson A.K. (1996). The Short Inflammatory Bowel Disease Questionnaire: A quality of life instrument for community physicians managing inflammatory bowel disease. CCRPT Investigators. Canadian Crohn’s Relapse Prevention Trial. Am. J. Gastroenterol..

[42] Hayes A.F. (2013). Introduction to Mediation, Moderation, and Conditional Process Analysis: A Regression-Based Approach.

[43] Hoyle R.H., Smith G.T. (1994). Formulating clinical research hypotheses as structural equation models: A conceptual overview. J. Consult. Clin. Psychol..

[44] Preacher K.J., Hayes A.F. (2008). Asymptotic and resampling strategies for assessing and comparing indirect effects in multiple mediator models. Behav. Res. Methods.

[45] Raftery A.E. (1995). Bayesian model selection in social research. Sociol. Methodol..

[46] Marusak H.A., Martin K.R., Etkin A., Thomason M.E. (2015). Childhood trauma exposure disrupts the automatic regulation of emotional processing. Neuropsychopharmacology.

[47] Varanoske A.N., McClung H.L., Sepowitz J.J., Halagarda C.J., Farina E.K., Berryman C.E., Lieberman H.R., McClung J.P., Pasiakos S.M., Karl J.P. (2022). Stress and the gut-brain axis: Cognitive performance, mood state, and biomarkers of blood-brain barrier and intestinal permeability following severe physical and psychological stress. Brain Behav. Immun..

[48] Spencer C., Mill R.D., Bhanji J.P., Delgado M.R., Cole M.W., Tricomi E. (2024). Acute psychosocial stress modulates neural and behavioral substrates of cognitive control. Hum. Brain Mapp..

[49] Décarie-Spain L., Hayes A.M., Lauer L.T., Kanoski S.E. (2024). The gut-brain axis and cognitive control: A role for the vagus nerve. Seminars in Cell & Developmental Biology.

[50] Barberio B., Zamani M., Black C.J., Savarino E.V., Ford A.C. (2021). Prevalence of symptoms of anxiety and depression in patients with inflammatory bowel disease: A systematic review and meta-analysis. Lancet Gastroenterol. Hepatol..

[51] Fracas E., Costantino A., Vecchi M., Buoli M. (2023). Depressive and anxiety disorders in patients with inflammatory bowel diseases: Are there any gender differences?. Int. J. Environ. Res. Public Health.

